# Supplementary material for: Genetically Modified Circulating Levels of Advanced Glycation End-Products and Their Soluble Receptor (AGEs-RAGE Axis) with Risk and Mortality of Breast Cancer
Source: Cancers (Basel). 2022 Dec 12;14(24):6124. doi: 10.3390/cancers14246124 (PMC9776370; doi:10.3390/cancers14246124)
Supplement: Supplementary file 1 [file cancers-14-06124-s001.zip › cancers-1967989-supplementary.pdf]

Supplementary Materials: Genetically Modified Circulating Levels of Advanced Glycation End-Products and Their Soluble Receptor (AGEs-RAGE Axis) with Risk and Mortality of Breast Cancer

Yu Peng †, Fubin Liu †, Yating Qiao, Peng Wang, Han Du, Changyu Si, Xixuan Wang, Kexin Chen * and Fangfang Song *

Table S1. Basic information of 14 SNP sites of the receptor coding genes involved in AGEs-RAGE axis.

Gene (Protein)	SNP locus	Allele	Consequence Type	MAF [1]	MAF [2]
AGER (RAGE)	rs2071288	C/T	Intron variant	0.01	0.02
	rs184003	C/A	Intron variant	0.14	0.16
	rs2070600	C/T	Missense variant	0.21	0.20
	rs1800624	A/T	Upstream Variant	0.17	0.15
	rs1800625	A/G	Upstream Variant	0.14	0.13
DDOST (AGE-R1)	rs607254	G/A	Intron Variant	0.28	0.32
	rs3738140	G/A	Intron Variant	0.10	0.08
	rs4704	G/A	Synonymous Variant	0.39	0.41
	rs10916846	T/C	Intron Variant	0.05	0.05
PRKCSH (AGE-R2)	rs11557488	G/A	Missense Variant	0.05	0.04
	rs160841	A/G	Intron Variant	0.12	0.10
LGALS3 (AGE-R3)	rs1009977	T/G	Intron Variant	0.26	0.28
	rs4644	C/A	Missense Variant	0.19	0.18
	rs4652	A/C	Missense Variant	0.40	0.39

[1] The minimum allele frequency was calculated according to the data of this study. [2] The minimum allele frequency was from National Center for Biotechnology Information. Abbreviations: AGEs, advanced glycation end-products; MAF, minimum allele frequency; RAGE, receptor for advanced glycation end-products.

Table S2. NRI and IDI of the combination of the risk factors and AGEs or sRAGE vs risk factors for predicting risk of breast cancer.

Variables	NRI (95% CI)	*P*	IDI (95% CI)	*P*
**Risk factors + AGEs**	0.11 (0.07-0.16)	<0.001	0.04 (0.03-0.05)	<0.001
**Risk factors + AGEs/sRAGE**	0.16 (0.12-0.20)	<0.001	0.06 (0.05-0.07)	<0.001

Risk factors: BMI, education, menarche age, menopause, estrogen replacement therapy, smoking, drinking, negative events, history of benign breast disease, breast cancer history of first-degree relative. Abbreviations: AGEs, advanced glycation end-products; BMI, body mass index; IDI, integrated discrimination improvement; NRI, net reclassification improvements; sRAGE, soluble receptor for advanced glycation end-products.

Table S3. The distribution of AGEs and sRAGE by clinicopathological features in breast cancer patients.

	AGEs (ng/ml)				*P*	sRAGE (pg/ml)				*P*
	Q1	Q2	Q3	Q4		Q1	Q2	Q3	Q4	
**Death**					0.028					0.834
No	122 (85.3)	212 (87.2)	236 (80.0)	264 (78.6)		259 (82.8)	231 (83.01)	190 (81.2)	154 (80.2)	
Yes	21 (14.7)	31 (12.8)	59 (20.0)	72 (21.4)		54 (17.3)	47 (16.9)	44 (18.8)	38 (19.8)	
**TNM Stage**					0.847					0.827
Early stage (0-IIA)	64 (60.4)	87 (64.4)	111 (59.7)	141 (61.6)		130 (61.0)	108 (59.3)	94 (64.4)	71 (61.7)	
Late stage (IIB-IV)	42 (39.6)	48 (35.6)	75 (40.3)	88 (38.4)		83 (39.0)	74 (40.7)	52 (35.6)	44 (38.3)	
**Molecular Subtype**					0.350					0.740
Basal	17 (11.9)	20 (8.2)	22 (7.4)	25 (7.4)		20 (6.4)	24 (8.6)	24 (10.3)	16 (8.3)	
Her2	30 (21.0)	41 (16.9)	50 (17.0)	59 (17.5)		61 (19.5)	49 (17.6)	34 (14.5)	36 (18.7)	
Luminal A	69 (48.2)	149 (61.3)	164 (55.6)	193 (57.3)		176 (56.2)	163 (58.4)	132 (56.4)	104 (54.3)	
Luminal B	27 (18.9)	33 (13.6)	59 (20.00)	60 (17.8)		56 (17.9)	43 (15.4)	44 (18.8)	36 (18.7)	
**Breast Density**					0.072					0.431
<25%	23 (18.2)	33 (14.9)	37 (15.2)	57 (21.0)		42 (16.3)	39 (16.5)	46 (22.3)	23 (14.0)	
25%-50%	68 (54.0)	97 (43.7)	113 (46.3)	117 (43.2)		122 (47.5)	110 (46.6)	93 (45.2)	70 (42.7)	
50%-75%	33 (26.2)	78 (35.1)	74 (30.3)	85 (31.4)		80 (31.1)	76 (32.20)	54 (26.2)	60 (36.6)	
>75%	2 (1.6)	14 (6.3)	20 (8.2)	12 (4.4)		13 (5.1)	11 (4.7)	13 (6.3)	11 (6.7)	
**Sex Hormones**										
Follicle stimulating hormone (FSH)	46.4 (11.3-77.7)	46.5 (11.7-70.8)	47.6 (11.8-67.7)	31.9 (8.2-61.8)	0.123	30.5 (7.6-63.2)	44.6 (11.7-68.2)	49.7 (21.1-71.7)	47.2 (9.4-69.0)	0.067
Estradiol (E2)	11.9 (7.6-61.5)	15.5 (7.8-54.6)	17.7 (8.4-51.8)	27.3 (13.1-83.1)	0.025	22.0 (10.6-79.3)	18.5 (7.8-74.8)	15.1 (8.1-39.5)	17.1 (8.1-54.2)	0.299
Prolactin	14.4 (8.4-20.1)	11.9 (8.7-19.3)	12.7 (9.2-18.7)	14.4 (10.0-21.1)	0.376	13.3 (9.8-20.3)	12.8 (8.5-18.4)	12.7 (9.9-17.8)	14.6 (8.9-22.7)	0.714
Progesterone	0.4 (0.2-0.7)	0.4 (0.3-0.6)	0.4 (0.2-0.6)	0.5 (0.3-0.8)	0.071	0.4 (0.3-0.7)	0.5 (0.2-0.8)	0.4 (0.2-0.6)	0.4 (0.3-0.6)	0.373

Testosterone	0.2 (0.1-0.3)	0.2 (0.1-0.3)	0.2 (0.2-0.3)	0.2 (0.1-0.3)	0.511	0.2 (0.1-0.3)	0.2 (0.1-0.3)	0.2 (0.1-0.3)	0.2 (0.2-0.3)	0.452
Luteinizing hormone (LH)	22.0 (14.4-34.5)	23.2 (7.8-33.2)	21.8 (9.2-32.6)	20.5 (6.0-31.0)	0.450	14.6 (4.8-30.1)	22.9 (8.2-32.5)	26.9 (14.4-35.1)	23.6 (10.9-33.8)	0.001

Abbreviations: AGEs, advanced glycation end-products; E2, estradiol; FSH, follicle stimulating hormone; LH, luteinizing hormone; PRL, prolactin; sRAGE, soluble receptor for advanced glycation end-products; TNM, tumor node metastasis.

Table S4. Correlations between AGEs, sRAGE and hormones.

	AGEs (ng/ml)		sRAGE (pg/ml)	
	**r**	***P***	**r**	***P***
**Follicle stimulating hormone (FSH)**	-0.111	0.021	0.084	0.083
**Estradiol (E2)**	0.130	0.007	-0.053	0.274
**Prolactin**	0.031	0.541	0.040	0.433
**Progesterone**	0.057	0.240	-0.051	0.292
**Testosterone**	0.044	0.364	0.011	0.817
**Luteinizing hormone (LH)**	-0.077	0.113	0.140	0.004

Abbreviations: AGEs, advanced glycation end-products; sRAGE, soluble receptor for advanced glycation end-products.

Table S5. Associations of genetic variants in AGEs-RAGE axis with risk and prognosis of breast cancer.

Gene (Protein)	SNPs	Genotype	Control	Case	*P*	P_{HWE}	OR (95%CI)	HR (95%CI)
			N (%)	*N* (%)				
AGER (RAGE)	**rs2071288**	CC	994 (97.8)	930 (97.7)	0.827	0.611	1.00 (reference)	1.00 (reference)
		CT	22 (2.2)	22 (2.3)			0.752 (0.369-1.533)	0.977 (0.353-2.699)
	**rs184003**	CC	767 (75.5)	689 (72.1)	0.236	0.042	1.00 (reference)	1.00 (reference)
		AC	236 (23.2)	253 (26.5)			1.113 (0.867-1.429)	0.944 (0.628-1.419)
		AA	13 (1.3)	13 (1.4)			1.113 (0.454-2.751)	0.907 (0.277-2.970)
	**rs2070600**	CC	630 (62.1)	577 (60.3)	0.230	0.151	1.00 (reference)	1.00 (reference)
		CT	338 (33.3)	347 (36.3)			1.177 (0.935-1.482)	0.819 (0.556-1.207)
		TT	46 (4.5)	33 (3.4)			1.125 (0.639-1.980)	1.061 (0.416-2.706)
	**rs1800624**	AA	697 (68.8)	655 (69.0)	0.783	0.486	1.00 (reference)	1.00 (reference)
		AT	292 (28.8)	268 (28.2)			0.912 (0.717-1.159)	1.125 (0.765-1.656)
		TT	24 (2.4)	27 (2.8)			0.884 (0.451-1.731)	2.259 (0.900-5.668)
	**rs1800625**	AA	733 (72.4)	722 (75.5)	0.288	0.777	1.00 (reference)	1.00 (reference)
		AG	256 (25.3)	216 (22.6)			0.857 (0.665-1.106)	1.266 (0.840-1.908)
		GG	23 (2.3)	18 (1.9)			0.909 (0.422-1.957)	0.741 (0.100-5.466)
DDOST (AGER1)	**rs607254**	GG	538 (53.1)	489 (51.2)	0.698	0.485	1.00 (reference)	1.00 (reference)
		AG	404 (39.8)	396 (41.4)			1.033 (0.824-1.296)	1.169 (0.807-1.693)
		AA	72 (7.1)	71 (7.4)			0.984 (0.646-1.500)	0.967 (0.475-1.970)
	**rs3738140**	GG	829 (81.8)	793 (81.2)	0.955	0.559	1.00 (reference)	1.00 (reference)
		AG	177 (17.4)	178 (17.9)			0.914 (0.690-1.211)	0.878 (0.536-1.438)
		AA	8 (0.8)	8 (0.9)			0.888 (0.261-3.028)	1.499 (0.351-6.404)
	**rs4704**	GG	385 (38.0)	337 (35.2)	0.412	0.212	1.00 (reference)	1.00 (reference)
		AG	488 (48.2)	475 (49.7)			1.005 (0.792-1.274)	1.114 (0.754-1.644)
		AA	140 (13.8)	144 (15.1)			1.005 (0.721-1.403)	0.964 (0.552-1.682)
	**rs10916846**	TT	917 (90.4)	843 (88.3)	0.120	0.020	1.00 (reference)	1.00 (reference)
		CT	97 (9.6)	112 (11.7)			1.210 (0.851-1.719)	0.937 (0.517-1.699)
PRKCSH (AGER2)	**rs11557488**	GG	908 (89.5)	863 (90.2)	0.283	0.967	1.00 (reference)	1.00 (reference)
		AG	102 (10.0)	93 (9.7)			0.966 (0.670-1.393)	1.320 (0.747-2.331)
		AA	5 (0.5)	1 (0.1)			0.078 (0.008-0.758)	/
	**rs160841**	AA	783 (78.1)	716 (76.7)	0.498	0.309	1.00 (reference)	1.00 (reference)
		AG	210 (21.0)	205 (21.9)			1.040 (0.796-1.360)	1.145 (0.744-1.762)
		GG	9 (0.9)	13 (1.4)			1.116 (0.375-3.323)	1.036 (0.313-3.427)
LGALS3 (AGER3)	**rs1009977**	TT	542 (53.5)	528 (55.1)	0.740	0.828	1.00 (reference)	1.00 (reference)
		GT	400 (39.4)	362 (37.8)			1.007 (0.800-1.267)	1.074 (0.742-1.555)
		GG	72 (7.1)	68 (7.1)			1.076 (0.709-1.631)	1.035 (0.487-2.199)
	**rs4644**	CC	663 (65.3)	637 (66.8)	0.207	0.353	1.00 (reference)	1.00 (reference)
		AC	326 (32.1)	282 (29.5)			1.014 (0.800-1.286)	1.225 (0.832-1.806)
		AA	26 (2.6)	35 (3.7)			1.430 (0.770-2.654)	1.180 (0.360-3.865)
	**rs4652**	AA	345 (34.0)	337 (35.4)	0.791	0.063	1.00 (reference)	1.00 (reference)
		AC	514 (50.6)	473 (49.7)			1.003 (0.790-1.274)	1.060 (0.715-1.572)
		CC	157 (15.4)	142 (14.9)			1.119 (0.804-1.558)	1.231 (0.714-2.125)

Covariates for OR: BMI, education, menarche age, menopause, estrogen replacement therapy, smoking, drinking, negative events, history of benign breast disease, breast cancer history of first-degree relatives. Covariates for HR: BMI, education, income, menopause, smoking, drinking, negative events, TNM stage, molecular subtype, cardiovascular disease, diabetes. Abbreviations: AGEs, advanced glycation end-products; BMI, body mass index; CI, confidence interval; HR, hazard ratio; OR, odds ratio; RAGE, receptor for advanced glycation end-products; SNPs, single-nucleotide polymorphisms; TNM, tumor node metastasis.

Table S6. Associations between SNPs in *DDOST*, *PRKCSH*, *LGALS3* and plasma AGEs levels.

Gene (Protein)	SNPs	Genotype	Case (N)	AGEs (ng/ml) Median (IQR)	*P*	Control (N)	AGEs (ng/ml) Median (IQR)	*P*
DDOST (AGER1)	**rs607254**	GG	489	6.5 (3.0-12.7)	0.842	538	2.6 (1.2-7.2)	0.816
		AG	396	6.1 (2.8-12.1)		404	2.7 (0.9-7.2)	
		AA	71	5.4 (2.4-11.1)		72	2.3 (1.2-8.5)	
	**rs3738140**	GG	775	6.1 (2.8-12.7)	0.368	829	2.5 (1.0-7.3)	0.477
		AG	171	7.8 (3.1-12.3)		177	3.4 (1.2-7.1)	
		AA	8	1.4 (0.7-7.8)		8	3.2 (1.5-4.4)	
	**rs4704**	GG	337	6.4 (3.1-13.1)	0.942	385	2.4 (1.1-6.9)	0.757
		AG	475	6.3 (2.7-11.9)		488	2.9 (1.0-7.5)	
		AA	144	6.1 (3.2-11.7)		140	2.8 (1.1-6.5)	
	**rs10916846**	TT	843	6.1 (2.8-12.6)	0.620	917	2.5 (1.0-7.3)	0.551
		CT	112	8.3 (3.4-12.3)		97	3.8 (1.3-6.8)	
PRKCSH (AGER2)	**rs11557488**	GG	863	6.2 (2.8-123)	0.539	908	2.5 (1.0-7.2)	0.336
		AG	93	6.9 (3.1-12.3)		102	3.2 (1.4-7.5)	
		AA	1	0.8		5	6.6 (2.3-27.3)	
	**rs160841**	AA	716	6.2 (2.8-11.9)	0.187	783	2.6 (1.1-7.1)	0.505
		AG	205	6.5 (3.2-13.6)		210	2.6 (1.1-7.9)	
		GG	13	5.1 (2.1-16.3)		9	1.6 (0.9-2.4)	
LGALS3 (AGER3)	**rs1009977**	TT	528	6.8 (3.0-13.7)	0.188	542	2.6 (1.0-7.1)	0.683
		GT	362	5.7 (2.7-11.1)		400	2.9 (1.1-7.4)	
		GG	68	5.2 (2.6-10.3)		72	2.2 (1.0-6.4)	
	**rs4644**	CC	637	6.7 (3.0-13.5)	0.296	663	2.6 (1.0-7.1)	0.674
		AC	282	5.3 (2.7-10.8)		326	2.9 (1.2-7.5)	
		AA	35	7.1 (2.7-13.4)		26	2.7 (1.1-5.7)	
	**rs4652**	AA	337	7.1 (3.1-13.3)	0.157	345	2.5 (1.0-7.2)	0.761
		AC	473	5.9 (2.7-11.9)		514	2.7 (1.1-7.4)	
		CC	142	5.4 (2.7-11.0)		157	2.5 (1.1-5.8)	

Abbreviations: AGEs, advanced glycation end-products; IQR, interquartile range; RAGE, receptor for advanced glycation end-product; SNPs, single-nucleotide polymorphisms.

Table S7. Association between *AGER* gene SNPs and sRAGE levels.

Gene (Protein)	SNP	Genotype	Case (N)	sRAGE(pg/ml) Median (IQR)	*P*	β(95%CI)	Control (N)	sRAGE(pg/ml) Median (IQR)	*P*	β(95%CI)
AGER	**rs2071288**	CC	930	686.4 (523.6-920.9)	0.213	ref	994	828.9 (608.4-1051.8)	0.064	ref
(RAGE)		CT	22	656.4 (467.9-782.8)		-100.7 (-246.5-45.1)	22	690.5 (492.7-984.7)		-97.4 (-83.5-61.1)
	**rs184003**	CC	689	678.7 (517.7-883.7)	0.043	ref	767	814.9 (591.6-1044.8)	0.121	ref
		AC	253	716.0 (530.7-985.3)		85.7 (36.9-134.5)	236	849.9 (653.4-1080.2)		46.6 (-5.6-98.8)
		AA	13	667.9 (574.7-917.3)		105.3 (-74.3-284.9)	13	862.7 (701.3-931.2)		14.3 (-178.4-207.0)
	**rs2070600**	CC	577	767.3 (612.2-1008.1)	<0.001	ref	630	910.2 (714.9-1132.7)	<0.001	ref
		CT	347	593.7 (452.5-762.9)		-209.5 (-251.2--167.9)	338	668.3 (517.2-892.8)		-232.1 (-275.0--189.1)
		TT	33	298.4 (190.0-432.7)		-484.3 (-596.3--372.3)	46	369.7 (274.9-428.7)		-568.6 (-664.9--472.3)
	**rs1800624**	AA	655	667.9 (500.2-894.0)	0.004	ref	697	786.9 (576.1-1021.8)	<0.001	ref
		AT	268	722.8 (562.2-970.7)		50.8 (3.0-98.6)	292	881.6 (665.8-1101.3)		89.6 (41.5-137.7)
		TT	27	772.0 (547.8-1008.1)		111.3 (-21.9-244.5)	24	992.1 (750.8-1230.0)		193.2 (53.6-332.8)
	**rs1800625**	AA	722	684.5 (517.7-906.7)	0.702	ref	733	815.3 (601.8-1049.8)	0.067	ref
		AG	216	684.1 (538.6-942.0)		5.6 (-46.6-57.8)	256	833.1 (628.7-1041.6)		7.2 (-43.8-58.3)
		GG	18	781.5 (639.0-909.8)		-24.8 (-178.6-129.0)	23	999.7 (735.5-1263.5)		171.9 (16.2-327.7)

Covariates: BMI, education, smoking, drinking, negative events. Abbreviations: CI, confidence interval; IQR, interquartile range; RAGE, receptor for advanced glycation end-products; sRAGE, soluble receptor for advanced glycation end-products; SNP, single-nucleotide polymorphism.

Table S8. Associations of *AGER* gene haplotypes with risk and prognosis of breast cancer.

**Haplotypes***	**Case**	**Control**	**OR (95%CI)**	**HR (95%CI)**
**CT**	276 (14.57)	305 (15.08)	ref	ref
**CA**	1209 (63.83)	1289 (63.75)	1.140 (0.914-1.421)	0.719 (0.529-0.979)
**TT**	45 (2.38)	35 (1.73)	1.537 (0.887-2.662)	0.498 (0.212-1.174)
**TA**	364 (19.22)	393 (19.44)	1.224 (0.938-1.597)	0.758 (0.519-1.106)

Covariates for OR: BMI, education, menarche age, menopause, estrogen replacement therapy, smoking, drinking, negative events, history of benign breast disease, breast cancer history of first-degree relatives. Abbreviations: BMI, body mass index; CI, confidence interval; HR, hazard ratio; OR, odds ratio; TNM, tumor node metastasis. *: alleles in order of rs2070600 (C/T) and rs1800624 (A/T).

Table S9. The association of *AGER* gene haplotypes and concentration of sRAGE.

**Haplotypes***	**N**	**sRAGE (pg/ml)**	β (95%CI)	***P***
**CT**	581	834.7 (642.2, 1075.5)	ref	
**CA**	2498	792.9 (603.7, 1027.6)	-36.5 (-66.7, -6.4)	0.018
**TT**	80	652.9 (526.4, 911.1)	-161.1 (-237.1, -85.1)	<0.001
**TA**	757	570.4 (400.3, 762.9)	-270.6 (-306.7, -234.4)	<0.001

Covariates: BMI, education, menarche age, menopause, estrogen replacement therapy, smoking, drinking, negative events, history of benign breast disease, breast cancer history of first-degree relatives. Abbreviations: BMI, body mass index; CI, confidence interval; OR, odds ratio; sRAGE, soluble receptor for advanced glycation end-products. *: alleles in order of rs2070600 (C/T) and rs1800624 (A/T).

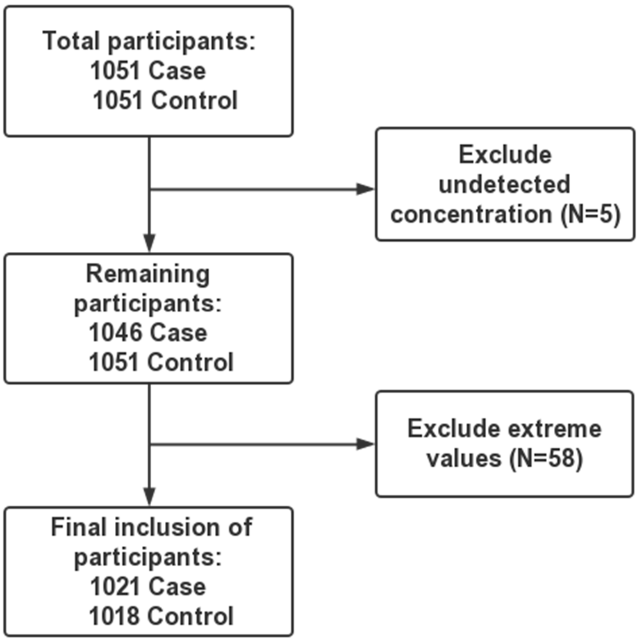

Figure S1. Flow chart of study population.

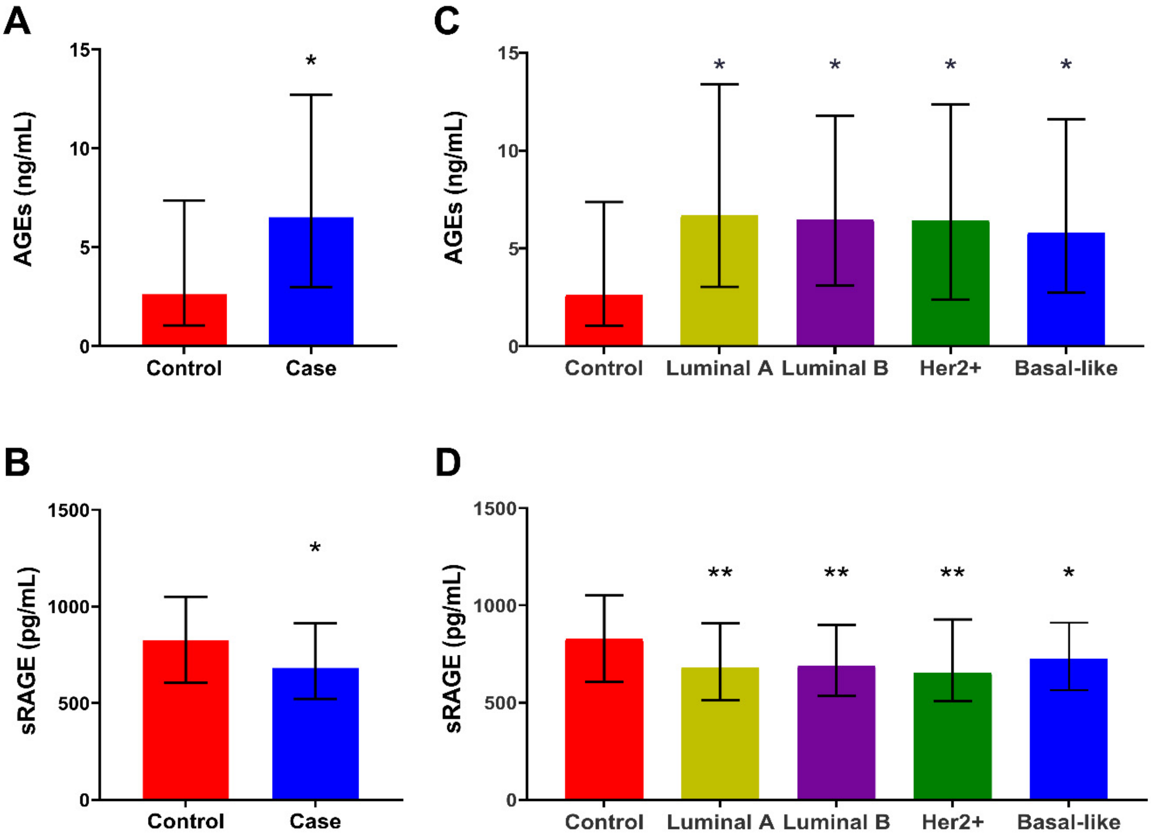

Figure S2. Distribution of AGEs and sRAGE in case-control population and molecular subtypes of breast cancer. *: Compared with the control group, $P<0.05$; **: Compared with the control group, $P<0.01$. Abbreviations: AGEs, advanced glycation end-products; sRAGE, soluble receptor for advanced glycation end-products.

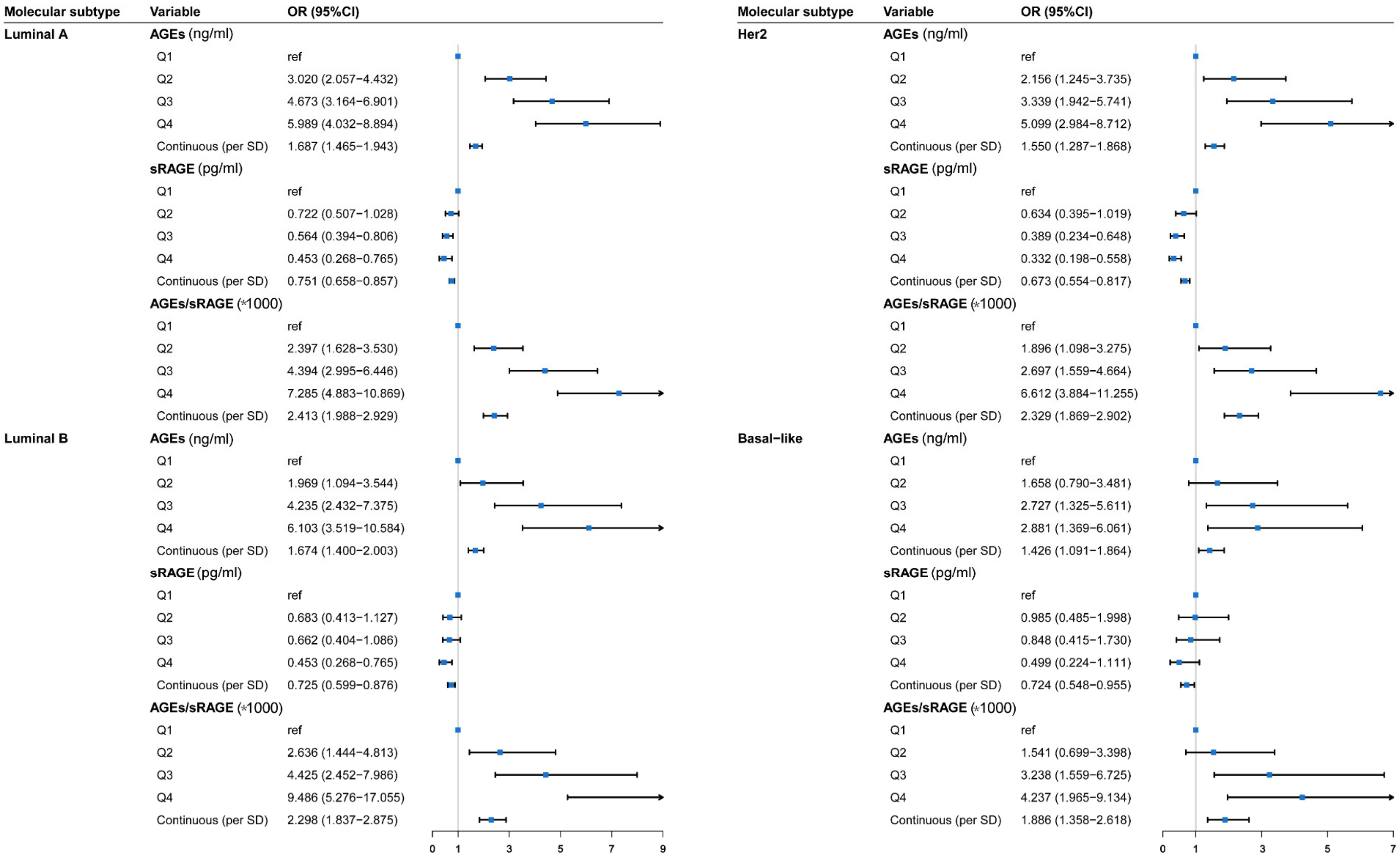

Figure S3. Quartile of AGEs, sRAGE and AGEs/sRAGE and risks of different breast cancer molecular subtypes. Covariates: BMI, education, menarche age, menopause, estrogen replacement therapy, smoking, drinking, negative events, history of benign breast disease, breast cancer history of first-degree relatives. Abbreviations: AGEs, advanced glycation end-products; BMI, body mass index; CI, confidence interval; OR, odds ratio; ROC, receiver operating curve; sRAGE, soluble receptor for advanced glycation end-products.

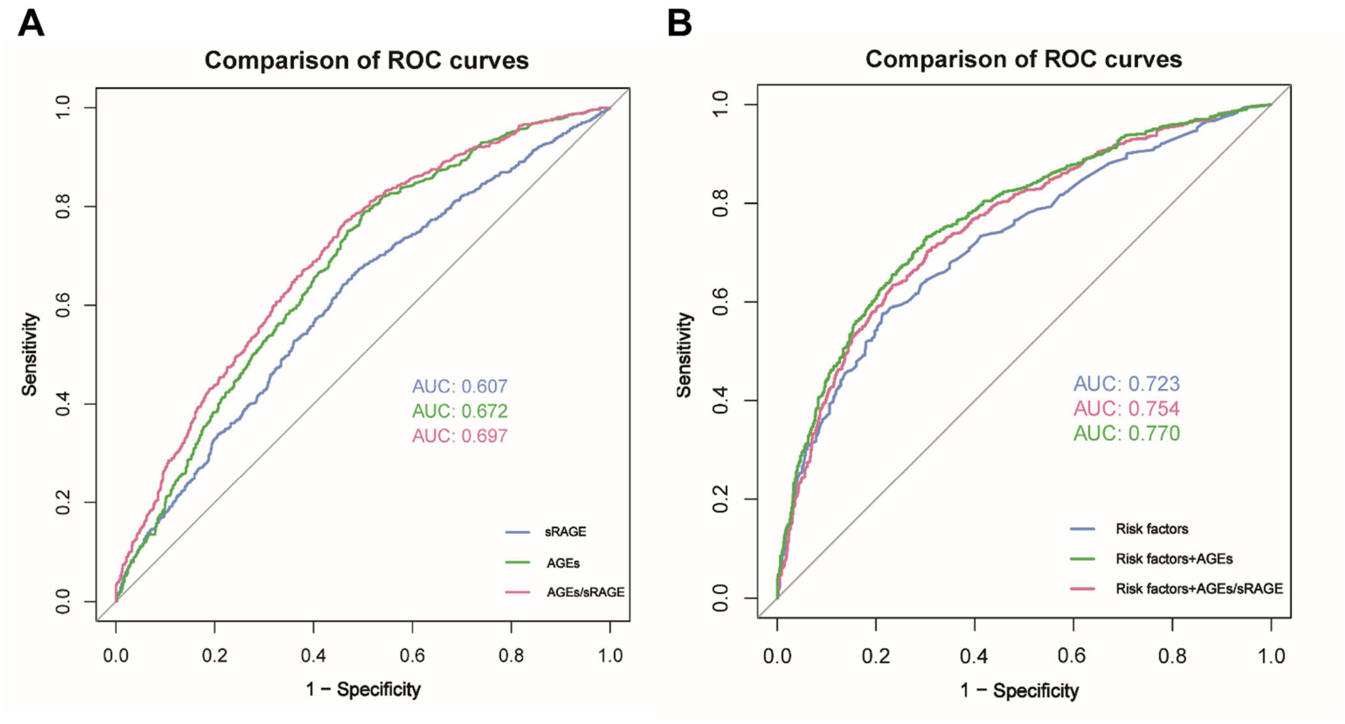

Figure S4. (A) ROC curves for AGEs, sRAGE and AGEs/sRAGE, (B) risk factors, AGEs or sRAGE and risk factors for breast cancer. Risk factors: BMI, education, menarche age, menopause, estrogen replacement therapy, smoking, drinking, negative events, history of benign breast disease, breast cancer history of first-degree relatives. Abbreviations: AGEs, advanced glycation end-products; BMI, body mass index; ROC, receiver operating curve; sRAGE, soluble receptor for advanced glycation end-products.

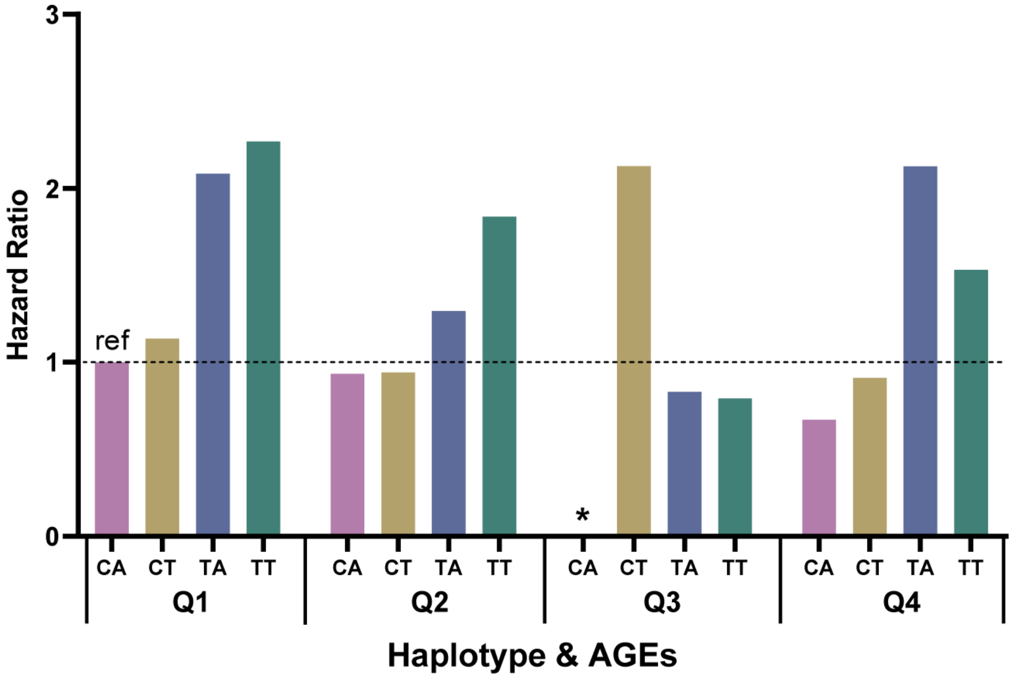

Figure S5. The interaction of *AGER* gene haplotypes with AGEs in breast cancer prognosis. Covariates: BMI, education, income, menopause, smoking, drinking, negative events, TNM stage, molecular subtype, cardiovascular disease, diabetes. Abbreviations: BMI, body mass index; CI, confidence interval; HR, hazard ratio; TNM, tumor node metastasis. All *P* values were not statistically significant. * Since there were no deaths, HR could not be calculated.
